# Detection of rabbit Haemorrhagic disease virus 2 during the wild rabbit (*Oryctolagus cuniculus*) eradication from the Berlengas archipelago, Portugal

**DOI:** 10.1186/s12917-017-1257-3

**Published:** 2017-11-15

**Authors:** F.A. Abade dos Santos, C. Carvalho, Oliveira Nuno, J. J. Correia, M. Henriques, M. C. Peleteiro, M. Fevereiro, M. D. Duarte

**Affiliations:** 1 Centro de Investigação Interdisciplinar em Sanidade Animal (CIISA), Faculdade de Medicina Veterinária, Universidade de Lisboa. Av. da Universidade Técnica, 1300-477 Lisbon, Portugal; 2 Instituto de Ciências Agrárias e Ambientais Mediterrânicas (ICAAM); Instituto de Investigação e Formação Avançada (IIFA), Universidade de Évora. Núcleo da Mitra, 7000 Évora, Portugal; 3 Sociedade Portuguesa para o Estudo das Aves (SPEA), Av. Columbano Bordalo Pinheiro, 87, 3º Andar, 1070-062 Lisboa, Portugal; 4 Instituto Nacional de Investigação Agrária e Veterinária (INIAV), Laboratório de Virologia. Av. da República, Quinta do Marquês, 2780-157 Oeiras, Portugal; 5 Rua Quinta do Pinto N°5 3°D, 2660-067 Loures, Frielas Portugal

**Keywords:** Rabbit haemorrhagic disease virus, RHDV2, Wild rabbit, *Oryctolagus cuniculus*, Berlengas, Berlenga Island, UNESCO

## Abstract

**Background:**

In the regular wildlife monitoring action carried out in the summer of the past few years at the Berlenga Island, wild rabbits (*Oryctolagus cuniculus*) have been repeatedly found dead. However, the origin of those deaths was never investigated. Our aim was to investigate the cause of death of 11 rabbits collected between April and May 2016.

**Results:**

While screening samples from rabbit carcasses for the major viral rabbit pathogens, five tested positive to RHDV2 but all were negative for RHDV and myxoma virus (MYXV). For six RHDV2-negative specimens, emaciation and parasitism were considered the most probable cause of death. Lesions identified in the RHDV2-positive rabbits included non-suppurative diffuse hepatic necrosis and pulmonary lesions varying from congestion and oedema of the lungs to interstitial pneumonia. Sequencing analysis of the *vp60* gene obtained from two specimens showed identical *vp60* sequences. Comparison with other known RHDV2 strains from public databases through BLAST analysis revealed a closer similarity with strains from Alentejo collected during 2013. Maximum Likelihood and Bayesian phylogenetic analysis showed that the 2016 strains from the archipelago have a higher resemblance with a group of strains mostly collected in the South of Portugal between 2013 and 2014.

**Conclusion:**

The results suggest that RHDV2 may have been introduced on the Berlenga Island a few years ago, having evolved separately from mainland strains due to insularity.

## Background

**Fig. 1:**
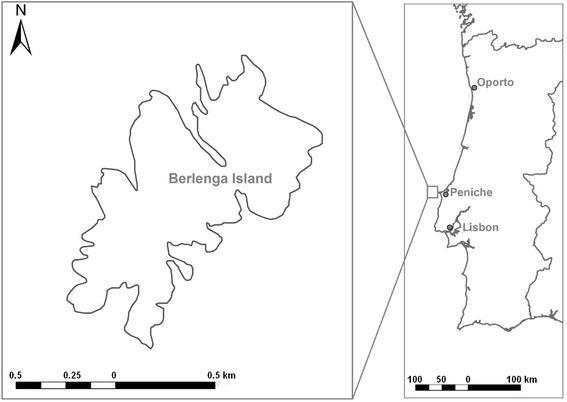
Berlengas Island, the major island of the Berlengas’ archipelago, located in the Portuguese maritime coast, and Peniche municipality (Portugal mainland) are marked on the map

The Berlengas (Fig. 1) is a small archipelago located 5.5 nautical miles (about 10 km) west off Peniche, a fishing town in the Portuguese Atlantic coast. It encompasses the Berlenga Island (1500 by 800 m long with a surface area of 80 ha), a group of small surrounding islets as well as two other more distant groups of islets (Estelas and Farilhões), being designated as Natural Reserve since 1981.

Berlenga is the only island that receives tourists, holding only a small group of houses used seasonally by local fishermen. During the fifteenth century, this island was a popular hunting royal province, probably due to the high population of European rabbits (*Oryctolagus cunniculus*), which exceptional abundance was reported already in 1465 [5].

**Fig. 2:**
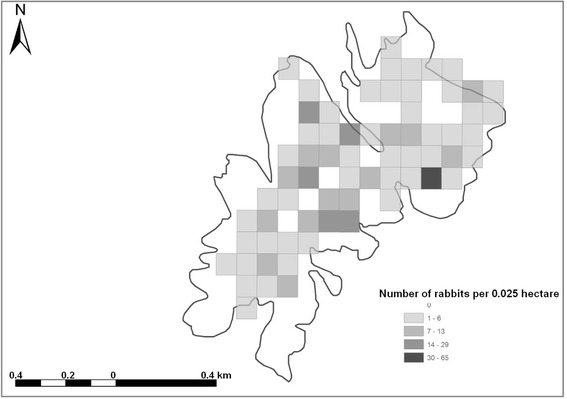
Density of the wild rabbit population in the Berlengas Natural Reserve calculated through several transects performed over 2015 and 2016

Presently, Berlenga is the only Island from this archipelago where wild rabbits are present, with a population size estimated at 38 to 133 individuals, calculated through several transects performed over 2015 and 2016 (Fig. 2) (Oliveira N, *unpublished data*). It is assumed that rabbits of unknown origin were brought onto this island for the first time during the fifteenth century [5]. Lighthouse keepers are known to have taken domestic rabbits into the island for backyard farming, on different occasions during the last two centuries. Those animals eventually escaped and mixed together with the remaining population [5]. Recent molecular data analysis has proved the presence of domestic genome on Berlenga Island’s rabbits (Oliveira N, *unpublished data*). Over the last six centuries, rabbits have deeply changed the soil dynamics thereby speeding up erosion due to their digging behaviour [5]. Also, the detrimental impact of *Rattus rattus* (Linneaus 1758) on the Berlengas ecosystem was recognized [5]. Those reasons are leading a current process to eradicate rabbit and black rat populations from the Berlenga Island.

In the last couple of years, an abnormal mortality of rabbits has been observed on the island by marine biologists that are regular visitors (Oliveira N, *personal communication*). However, neither infectious pathogens nor toxicological agents were ever investigated to determine the origin of those deaths. Furthermore, to the best of our knowledge, no serological or pathogen surveys have been conducted in the past on the rabbit population of the island. The only available data originates from a study on the ecology of rabbits and black rats (*Rattus rattus*) ectoparasites [54].

Among the pathogens that most severely affect rabbit, myxoma virus (MYXV) [6, 28, 57] and rabbit haemorrhagic disease virus (RHDV) [40] assume a major relevance and their rate of transmission in the wild is affected by fluctuations in the wild rabbits’ population density [30, 32].

Myxoma virus (MYXV), a large dsDNA virus, is a member of the *Leporipoxvirus* genus of the family *Poxviridae,* subfamily *Chordopoxvirinae*, [35]. It appears to be passively transmitted to European and American rabbits (*Sylvilagus brasiliensis* and *S. bachmani*) as well as to hares (*Lepus europaeus*) through the biting of insects that act as mechanical vectors, once the virus adheres to their mouthparts [35, 34, 12]. In its natural host (*S. brasiliensis*) and in hares, MYXV rarely causes disease [35], but European domestic and wild rabbits may develop a rapid systemic infection causing death in few days [35].

Rabbit haemorrhagic disease (RHD) is a highly contagious infectious disease of the European wild and domestic rabbits, caused by a virus of the *Lagovirus* genus, family Caliciviridae. RHD is characterised by high morbidity and mortality: 70–90% for RHDV/RHDVa and 5–70% for RHDV2 [52]. The disease was first identified in 1984 in China [40]. In Europe, RHD was first diagnosed in Italy in 1986 [15], soon becoming endemic in several countries [2]. In the Iberian Peninsula, from where European rabbits originated and where they are key species of the ecosystem [21], the first outbreaks occurred in 1988 in Spain [7] and in the following year in Portugal (Anonymous, 1989)[2], causing severe reduction of the wild rabbit populations (Villafuerte et al., 1995)[2] and considerable economic losses in the rabbit industry [7]. Despite the low level of genetic variation found, the molecular characterization of RHDV strains allowed the distinction of six well-defined phylogenetic genogroups (G1 to G6) [51, 36].

In 2010, a new virus designated RHDV2 emerged with a distinct genetic and antigenic profile [38]. It was first identified in France in 2010 [38], and rapidly spread to several European countries including Italy [37], Spain [20], Portugal [1], the United Kingdom [64] and Scotland [9]. Outside the European continent, RHDV2 was first detected between late 2014 and early 2015, in the Azores archipelago [22]. More recently, RHDV2 was also reported in Australia [31], Finland (http://www.oie.int/wahis_2/public/wahid.php/Countryinformation/Countryreports) and North of Africa [44].

RHDV2 differs from RHDV in the clinical characteristics of the induced disease in terms of duration, mortality rates and the more frequent occurrence of subacute/ chronic forms [37]. Furthermore, RHDV2 also affects young rabbits with less than two months old, often before they leave the burrows, as well as RHDV vaccinated rabbits that, although protected against classical strains, are susceptible to RHDV2 infection [38, 20, 37]. In addition, RHDV2 is also able to infect hosts other than rabbits as the virus was detected in cape hares (*Lepus capensis*) [55] as well as Italian hares (*Lepus corsicanus*) [14].

In Portugal, since RHDV2 cases were found [1], the former circulating classical RHDV genogroups, mostly G1-related strains in mainland [49, 3] and G5 in Azores [25], were no longer identified, suggesting that RHDV2 replaced the classical RHDV strains probably due to a selective advantage of this new virus by overcoming the existing immunity to older strains [41].

In this study, we aimed to investigate the cause of death of wild rabbits on the Berlenga Island, occurring in the spring of 2016.

## Methods

### Sample collection

The rabbits used in this study were collected on the Berlenga Island, Portugal, between April and May 2016, within the scope of the LIFE Berlengas project ("Conserving threatened habitats and species in Berlengas SPA through sustainable management", LIFE13 NAT/PT/000458).

Eleven cadavers, consisting of seven females (64%) and four males (26%), were selected from a larger group, based upon their good preservation status. All collected specimens were weighed. Age was estimated upon tarsus and skull length (occipitonasal length), measured with a micrometre. Skull length was defined as the highest distance from the cranial extremity of the premaxillae (excluding the incisors) to the rear of the occipital crest. This age estimation was determined according to the equation first used by Southern [60] and revised by Dunnet (1956) [27]. Prior to freezing, liver samples were collected. All cadavers were then frozen at −20 °C until necropsy.

### Virological examination

At the National Reference Laboratory (INIAV), rabbit pathogens associated with high mortality rates, namely RHDV, RHDV2 and MYXV, were investigated. Liver samples from the 11 specimens were homogenised with phosphate buffered saline (PBS) and clarified at 3000 g for 5 min. DNA and RNA were extracted from 200 μl of the clarified supernatant, corresponding to approximately 50 mg of tissue, in a BioSprint 96 nucleic acid extractor (Qiagen, Hilden, Germany) according to the manufacturer’s instructions. Samples were tested for RHDV2 by a specific RT-qPCR [23]. Screening for RHDV [62] was performed by sequencing of the amplicons obtained with primers RC9F and RC10R [62]. Conventional RT-PCR and RT-qPCR were performed with the One Step RT-PCR kit (Qiagen, Hilden, Germany).

The presence of myxoma virus was investigated by qPCR [26], using the FastStart TaqMan Probe Master Kit (Roche, Roche Diagnostics GmbH, Manheim, Germany).

For the real-time PCR systems described, undetectable Cq or Cq values >40 were considered negative.

### Nucleotide sequencing analysis

Amplification of the complete *vp60* sequences of RHDV2 strains was accomplished with two pairs of primers, 27F (5′-CCATGCCAGACTTGCGTCCC-3′) and 986R (5′-AACCATCTGGAGCAATTTGGG-3′), 717F (5′-CGCAGATCTCCTCACAACCC-3′) [24], and RC10R [62] enabling the obtainment of two overlapping fragments. Both 717F and 986R are specific for RHDV2. The One Step (Qiagen, Hilden, Germany) kit was used, under the manufacturers’ recommendations. Sequencing was accomplished using the BigDyeTM Terminator cycle sequencing kit (Applied Biosystems, Foster City, CA, USA).

The complete *vp60* nucleotide sequences of two RHDV2 strains (GenBank accession numbers KY247124 and KY247125) were determined on an automated 3130 Genetic Analyzer system (Applied Biosystems, Foster City, CA, USA).

### Phylogenetic analysis

The phylogenetic relationships between two RHDV2 *vp60* sequences from the Berlenga Island with other RHDV2 sequences originated in Portugal mainland, Azores and worldwide, were investigated. Sequence KC345614 (from a classical RHDV strain belonging to genogroup G5) was chosen as outgroup to root the trees.

Multiple alignments of the nucleotide sequences were generated by CLUSTAL Omega [58].

Phylogenetic inference was performed by Maximum Likelihood (ML) and Bayesian methods. For ML analysis, the appropriated substitution model was determined resourcing to R software (R Development Core Team, 2011). The GTR model [61] with gamma-distributed rate variation across sites (GTR + G) showed the lowest BIC and AICc values and was subsequently used to infer phylogenetic relationships. Robustness of the tree nodes was assessed by bootstrapping 1000 times.

For the Bayesian analysis, the CLUSTAL Omega results were converted to the NEXUS format using Mesquite software [42] The phylogenetic tree was obtained with a Bayesian inference of phylogeny throughout the MrBayes version 3.1.2 software that uses the Markov chain Monte Carlo simulation technique to approximate the posterior probabilities (pp) of trees [33, 56]. MrBayes analysis was performed using the GTR model (nst = 6) with gamma-shaped rate variation with a proportion of invariable sites (rates = invgamma). The analysis was run for 10^6^ generations (ngen = 10^6^) with four chains of temperature (nchains = 4), and each chain was sampled every 10th generations (samplefreq = 10).

The graphical representation and edition of the phylogenetic trees were performed with FigTree v1.3.1 (http://tree.bio.ed.ac.uk/software/figtree/).

### Necropsy and histopathological examination

Necropsy and histopathological examination was carried out the Pathology Laboratory of the Faculty of Veterinary Medicine of the University of Lisbon.

Organs collected from necropsy (liver and lungs) were submitted to fixation in 10% buffered formalin and processed for routine histopathological analysis. Sections were stained with H&E and Pearls Blue and microphotographs were obtained with a DP23 Olympus digital camera.

## Results

### Morphometric data

Weight of the 11 selected rabbits varied between 330 and 600 g (10 g sensitive scale). Mean tarsus and skull length was 44 mm and 75 mm, respectively corresponding to an estimated age of 43 to 83 days. All RHDV2-positive rabbits were less than six months old.

### Virological examination

Six liver samples were negative to RHDV2 and RHDV. The other five tested positively to RHDV2 by RT-qPCR [23]. None of the eleven rabbits showed cutaneous myxomas or were positive to myxoma virus by qPCR.

Sequencing of the complete *vp60* gene obtained from two specimens confirmed the presence of RHDV2 circulating in the island. These *vp60* sequences (KY247124 and KY247125) are identical (100% similarity).

### Phylogenetic analysis

Blast analysis of the Berlengas’ *vp60* sequence showed higher similarity (99%) with strains from the South of Portugal mainland, in particularly with strains from Barrancos obtained in 2013.

**Fig. 3:**
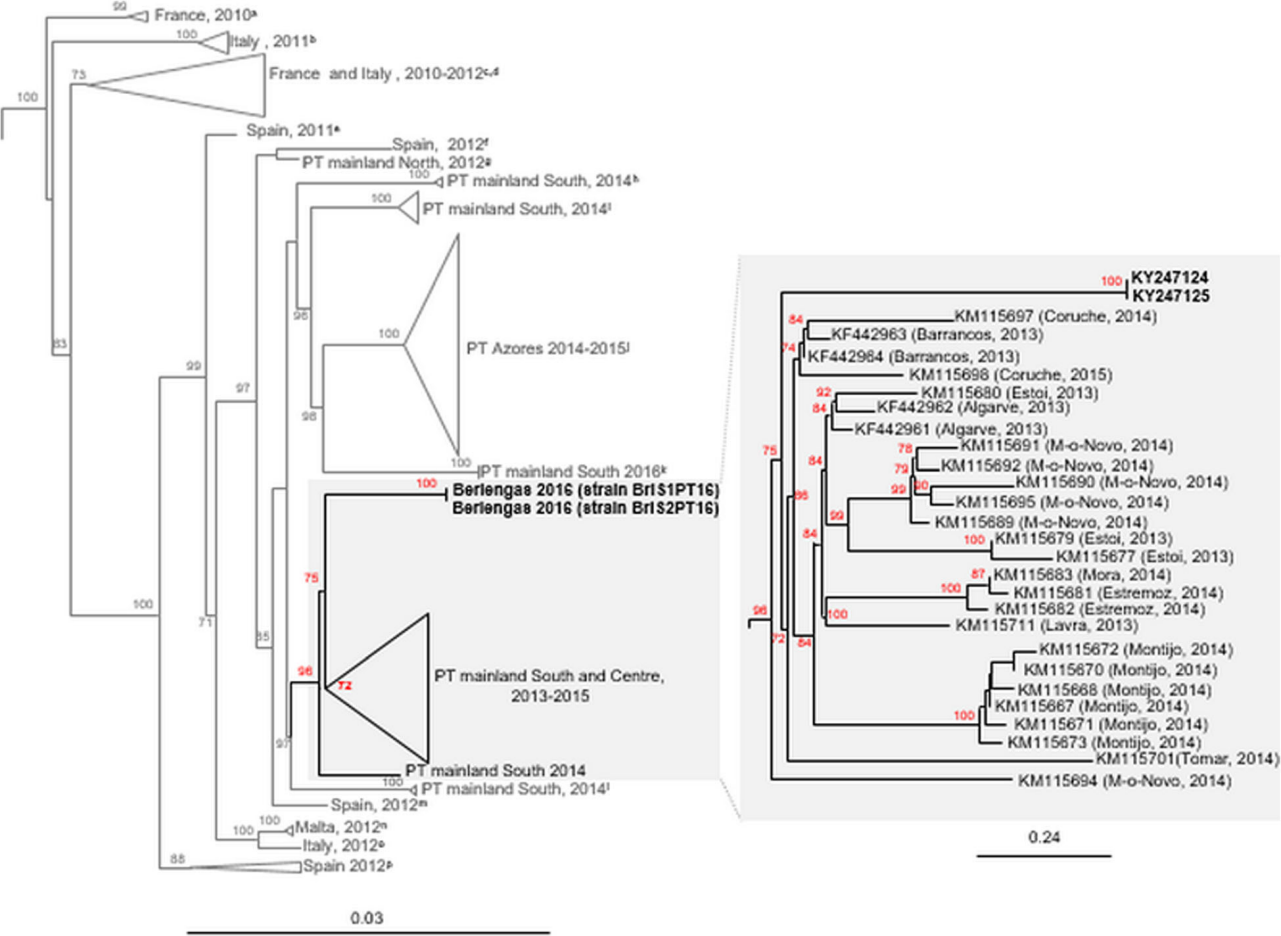
Maximum Likelihood (ML) phylogenetic tree of the RHDV2 *vp60* complete nucleotide sequences from the Berlenga island (2016) and others originated in Portugal mainland, Azores, Spain, France, Italy and Malta, available in Genbank. Bootstrap values (BS) are shown next to the nodes if equal or greater than 70. Sequence KC345614 (*not displayed*) was chosen as outgroup to root the tree. In the left side tree, the major groups are collapsed to facilitate visualization. a-HE800531, HE819400; b-JX106023, KC345611–12; c-HE800529, HE800530, HE800532 and FR819781; d-JQ929052, KC907712 and JX106022; e-KM87868; f-KP129395; g-KM979445; h-KM115675–76; i-KM115712–13; j-KT000295, KT000303, KT000308, KT000311, KT000316–319, KT000322–325 KT000327, KT000329–330, KT000332–333, KT000336, KT000339 and KT000341–343; k-KX132812 and KX132813; l-KM115684–5; m-KP129396; n-KJ957809 and KJ957810; o-KC741409; p-KP129397 and KP129399. “M-o-Novo” refers to the municipality of Montemor-o-Novo. A close-up of the RHDV2 strains more closely related with the strains from the Berlenga Island is shown in the right side of the figure

ML (Fig. 3) and Bayesian (*not* shown) trees were consistent, showing that the strains from the Berlenga Island are most closely related (Bootstrap value of 75) with a major group of strains originated mostly in the South between 2013 and 2014. No clear resemblance was noticed with any strain collected more recently.

### Necropsy and histopathological examination

The six RHDV2-negative rabbits, all aged less than six months, showed severe emaciation with reduced fat stores and muscle mass. The hair coat was in poor condition (rude and dull) and faecal material was adherent to the perineum. At necropsy, macroscopic lesions were neither observed in the liver nor in any of the other organs except for the small intestines that were distended, with inflammation and oedema of the jejunum and ileum. No bleedings or mucosal ulcerations were however found. In the fecal smear of the jejunum and ileum contents *Eimeria* spp. oocysts were observed (*results not shown*).

Four of the five RHDV2-positive rabbits showed low body condition (score 1 in the 1 to 5 in body condition scoring for rabbits, according to [53]). In all these specimens the noses were soiled by bloody discharge, and in three rabbits fluid faecal material in the perianal region was present. Organ preservation was considered good enough to assure lesions identification. Changes were mostly restricted to the respiratory apparatus and liver. Respiratory lesions, present in all rabbits, included congestion of the trachea, which contained serohaemorrhagic fluid, and congestion of the lungs. No signs of overt haemorrhage were detected. In all cases, there was discoloration and diminished consistency of the liver. Three rabbits, the same with soiled perianal region, showed distended caeca and had unsolidified faecal material in the colon. The fresh faecal smear analysis of the jejunum and ileum revealed the presence of *Eimeria* spp. oocysts.

**Fig. 4:**
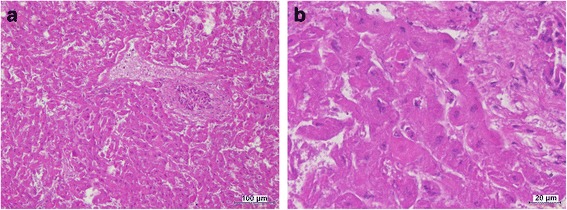
Liver of rabbit positive to RHDV2. **a** - Diffuse hepatocyte coagulation necrosis with no pattern distribution. Most hepatocytes are karyolytic and only very few display karyorrhexis or pyknosis. **b** – Detail of A showing necrotic hepatic cells dysplaing karyorrhexis and pyknosis (H&E)

**Fig. 5:**
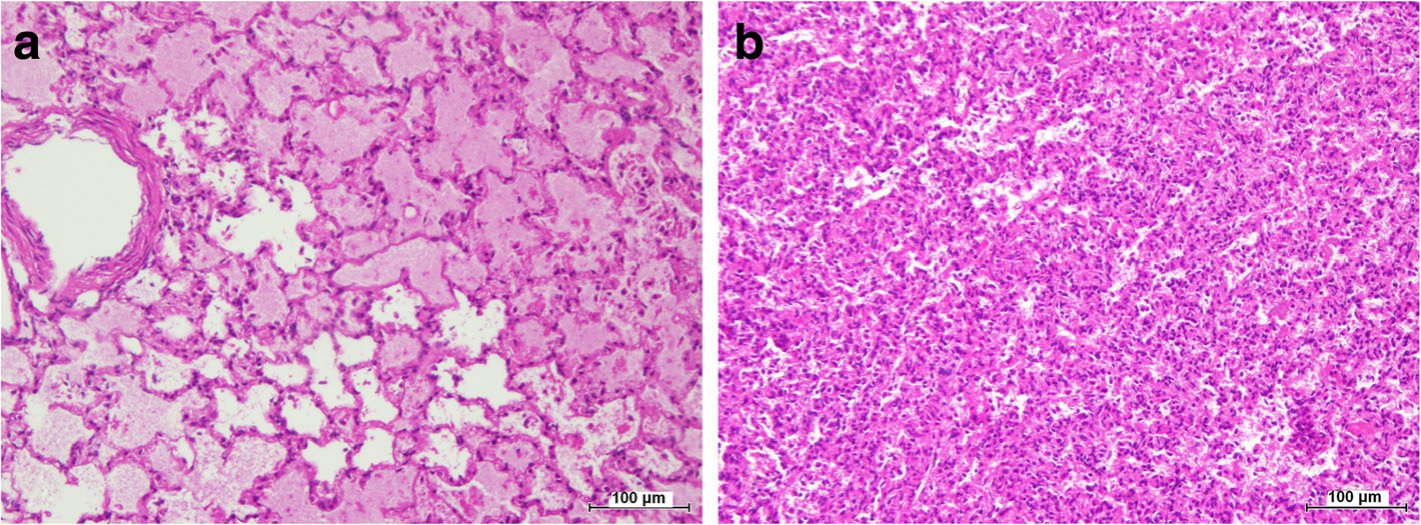
Lung of rabbits positive to RHDV2. **a** – Alveolar congestion and oedema. **b**-Interstitial pneumonia with inflammatory infiltrates in the alveolar septa by mononuclear cells, which are also present in the alveolar lumen (H&E)

Histology analysis of the liver showed diffuse hepatocyte coagulation necrosis with no pattern distribution, supporting a diagnosis of diffuse non-suppurative acute hepatic necrosis. Most hepatocytes were karyolytic and only very few displayed karyorrhexis or pyknosis (Fig. 4a and b). In one case, the necrotic hepatic cells revealed vacuolated profile. Iron pigment deposition in Kuppfer cells was regularly seen. Lung lesions varied from congestion and alveolar oedema (*n* = 2) to inflammatory infiltrates in the alveolar septa by mononuclear cells with atelectasia and inflammatory cells in the alveolar lumen, consistent with interstitial pneumonia (*n* = 3) (Fig. 5a and b).

## Discussion

Notwithstanding rabbit mortality at the Berlenga Island had not been investigated in previous years, our study clearly shows that RHDV2 was circulating in the island, at least since May 2016, and caused the death of five of the eleven rabbits investigated that died during the last spring. It is known that limited food availability and predation by dogs are also a cause for rabbit mortality in the island. Also, given the scarcity of fresh water in the island, the rabbit population of the Berlenga is highly dependent on the rainfall regime, with the mortality rate increasing during the dry months [63]. Despite no detailed parasitological examination was carried out during this study, the five RHDV2-negative young found in April, presented signs of intestinal coccidiosis along with emaciation that could have contributed to their deaths. Also, apart from those findings, no lesions were found in the liver and lungs that could suggest haemorrhagic disease. The impact of coccidiosis, caused by *Eimeria* spp., in the European rabbit populations of the Iberian Peninsula was recently evaluated in two ecological regions of Spain, estimating the coccidian prevalence by the mean oocyst excretion levels detected in faeces. Although in both areas the oocyst per gram of faeces was generally low, six *Eimeria* species were identified [59]. Among those (*E. coecicola, E. perforans, E media, E. magna, E. irresidua and E. flavescens*), *E. flavescens* is considered highly pathogenic for rabbits [39]. Curiously, the abundance and loads of Coccidia and nematodes in reintroduced rabbit populations in Spain showed no clear pattern with rabbit haemorrhagic disease prevalence [10]. Nonetheless, the impact of coccidea infection on morbidity and mortality of wild rabbit populations of the Berlenga Island was never investigated.

Myxoma virus-DNA was not detected in any of the 11 rabbits tested. Furthermore, none of these animals exhibited skin lesions, oedema or signs of conjunctivitis suggestive of nodular myxomatosis disease. Also, the qPCR developed by Duarte et al. [26] has allowed to detect MYXV-DNA in several organs of wild rabbits (*results not published*, Duarte et al.), which increases the confidence on the MYXV-PCR negative results obtained in the liver samples during this study.

RHDV2 infection was the cause of death of five rabbits from Berlenga Island collected in May 2016. However, given the reduced size of our sample, the positivity percentage of 45.5% (5/11 animals) is, most likely, merely indicative of the true mortality induced during the outbreak.

The characterization of RHDV2 induced lesions is still limited compared with that regarding RHDV. Post-mortem examination of RHDV2-infected rabbits revealed macroscopic lesions consistent with haemorrhages in several organs including heart, trachea, thymus, lungs, liver, kidneys, and gut, as well as jaundice [20, 24]. The liver appearance was described as soft and pale [24]. Histopathologic descriptions refer to haemorrhagic pneumonia and tracheitis, congestion of the liver and diffuse necrotizing hepatitis. Areas of focal necrosis were also described in the intestinal villi in the small intestine [20].

In the present report, lung lesions varied from simple congestion and alveolar oedema, indicating an acute evolution, to interstitial pneumonia suggesting a longer disease process. Haemorrhagic lesions were neither identified in the lungs nor in other organs, although rabbits consistently showed soiled noses, probably by blood tinged oedematous lung fluid. Interstitial pneumonia observed in three out of five rabbits have not been reported before in the post-mortem examination of RHDV or RHDV2 infected rabbits [43, 46, 24, 41]. It is possible that pneumonia could have eventually be due to a longer disease evolution or to bacterial infections occurring prior to the contact with the RHDV2. Inflammatory infiltrates by mononuclear cells are quite compatible with viral infection (Fig. 5b).

In fact, the diffuse coagulation necrosis registered in the liver of all rabbits is only compatible with an acute or hyperacute course the disease possibly favoured by the low body condition of the rabbits. The type of necrosis with most of the cells karyolitic and without a defined pattern distribution is more in agreement with the lesions described in European Brown Hare Syndrome (EBHS) [43], although the reports of RHDV and RDHV2 infected rabbits consistently refer to severe necrotic liver lesions [43, 46, 24, 41].

In summary, death of the infected rabbits analysed in this study must have been due to acute hepatic failure with lung congestion and oedema occurring closer to death. The relevance of the interstitial pneumonia remains unclarified as no other infectious agents were investigated apart from the aforementioned leporid-specific viruses. The fact that the cadavers were not fresh limited the confidence and significance in any bacterial identification.

The phylogenetic results revealed that the 2016 strains from the Berlenga Island formed an independent cluster in closer proximity with a group of sequences obtained between 2013 and 2014, mainly in the South of Portugal mainland. The higher resemblance with a group of older strains may suggest that RHDV2 was not introduced recently in the Island. Instead, and with accordance with the mortalities observed in the last years, RHDV2 introduction may have taken place a few years ago, circulating since then in the island. Because no intermediate strains between the Barrancos 2013 and Berlengas 2016 strains were found until the moment (*results not shown*), this possibility remains only hypothetical. Yet, the island geographic location may well have provided the necessary isolation for the Berlenga Island strains to evolve apart from other haplotypes that have been characterized in the latest years in Portugal mainland [1] [16] and in Azores [24, 4]. Due to the advanced stage of rabbit eradication at the time samples were collected, no serologic study could be carried out which may have allowed to clarify if RHDV2 was introduced on the island prior the 2016 outbreak.

Given the island eco-geographic particularities, RHDV2 introduction may have occurred by several routes. Beside human means and mechanical action of arthropod vectors [8, 19, 45], black rat may also have accounted for the viral introduction. Native from the Indian peninsula [50], the black rat is worldwide distributed [11] and is the only rodent species found on the island [5]. Although never demonstrated, similarly to *Apodemus silvaticus* and *Mus spretus* [48], black rats from RHDV2 infected areas may have carried the virus to the rabbit population of Berlengas after being inadvertently transported by the boats that arrive at the island. Other possibilities concern resident birds of prey such as the Common Kestrel (*Falco tinnunculus*) and the Peregrine Falcon (*Falco peregrinus*) or vagrant birds as the Common Buzzard (*Buteo buteo*), that may also have carried contaminated leftovers from RHDV2 infected rabbits from Portugal mainland. Furthermore, Berlenga Island holds a large population of Yellow-legged Gulls (*Larus michahellis*), estimated near 13,150 individuals [47], which daily feed on waste treatment plants, farms and aviaries on the surrounding grounds of the Peniche municipality (located in mainland and which also includes the Berlengas archipelago) [18]. No reliable data could be gathered regarding the occurrence of haemorrhagic disease in domestic rabbits in Peniche. However, abnormal mortality rates have been observed in wild rabbits from local game reserves, Atouguia da Baleia, Ferrel and Serra d’el-Rei, during the last years. RHDV2 infections have been demonstrated in most of the national territory since its introduction in 2012, drastically reducing the wild rabbit population in particular areas [1, 4, 24, 41]. Therefore, it is highly likely that the three Peniche neighbouring parishes were the game reserves are sited may have also been affected. The Berlenga Island location near the coast, support the hypothesis that the virus strains found in the island may have been introduced from the nearby RHDV2 affected geographical area. Direct contact with rabbit contaminated materials is therefore likely to have occurred since Yellow-legged Gulls are commonly seen transporting dead corpses of several animals into the island including parts of rabbits (Oliveira N., *personal communication*).

The exceptional terrestrial insular ecosystem of Berlengas encompasses unique characteristics [17]. The archipelago provides nesting conditions for seabird species from the families *Procellariidae*, *Hydrobatidae*, *Phalacrocoracidae*, and *Laridae*, such as the Cory’s Shearwater (*Calonectris borealis)*, the Band-rumped storm-petrel (*Hydrobates castro)* only present in Farilhões, the European Shag (*Phalacrocorax aristotelis)*, the Yellow-legged Gull and the Lesser Black-backed Gull (*Larus fuscus*) [17]. Cory’s Shearwater, Band-rumped Storm-petrel and European Shag are classified as vulnerable by the Portuguese RedList Book [13]. The role of rabbits on deviation of the original ecology of the Berlenga Island is controversial. Besides the effect of rabbits, it is known that the decline of the unique endemic vegetation such as the *Armeria berlengensi*, *Herniaria berlengiana*, *Pulicaria microcephala* as well as the *Lobularia maritime,* and *Frankenia laevis,* is also a consequence of the acid excrements of the overpopulated Yellow-legged Gull and competition by Ice plant (*Carpobrotus edulis*)*,* an invasive plant native to South Africa brought into the island in the 1980’s and which threatens its natural vegetation biodiversity [29].

The current conservation policy of the Berlengas archipelago biological heritage, classified as of high interest, is due to its protected habitats and vulnerable surrounding marine ecosystem, one of the richest Portuguese seawaters for which it was considered Nature Reserve by UNESCO since 2010. Aiming to guarantee the survival of several endangered species, a national plan to eradicate the rabbit and black rat population from Berlenga is in course. If successful, this study may have been the last opportunity to trace the RHDV2 passage through the Berlengas archipelago.

## Conclusions

Our results showed that the mortality observed in wild rabbits from the Berlenga Island during the spring of 2016 was due, at least in part, to RHDV2. Despite marine biologists working in the island have observed rabbit mortality in the last years, this study provides the first evidence of the circulation of RHDV2 in the Berlengas archipelago. Phylogenetic analysis showed that the RHDV2 strain circulating in Berlenga in 2016 is distinct from mainland Portugal strains, possibly due to geo-segregation, and more closely related with strains from the South of the country.

## Abbreviations

BLAST: Basic local alignment search tool
cq: Quantification cycle
DNA: Deoxyribonucleic acid
GTR: General time reversible
H&E: Hematoxylin and eosin
INIAV: Instituto Nacional de Investigação Agrária e Veterinária
ML: Maximum likelihood
MYXV: Myxoma virus
PBS: Phosphate buffered saline
RHD: Rabbit haemorrhagic disease
RHDV: Rabbit haemorrhagic disease virus
RHDV2: Rabbit haemorrhagic disease virus 2
RNA: Ribonucleic acid
RT-PCR: Reverse transcription polymerase chain reaction
RT-qPCR: Quantitative reverse transcription PCR
vp60: Major capsid protein
